# Therapeutic Outcomes of Janus Kinase Inhibitors in Refractory Nail Lichen Planus: A Systematic Review

**DOI:** 10.7759/cureus.108953

**Published:** 2026-05-16

**Authors:** Sultan Aqeel, Hassan Hannani, Mohammed E Mojiri, Faisal A Otaif, Ghena M Alsaadi, Abdulmajeed A Alghamdi, Fares F Alammari, Suliman K Alkhudairy, Hamza A Alzahrani, Mohammed M Akkam

**Affiliations:** 1 Dermatology, King Fahd Central Hospital, Jazan, SAU; 2 Medicine, Jazan University, Jazan, SAU; 3 General Practice, Jazan Health Cluster, Jazan, SAU; 4 Medicine, King Abdulaziz University, Jeddah, SAU; 5 Medicine, University of Bisha, Bisha, SAU; 6 Medicine, Almaarefa University, Riyadh, SAU; 7 Medicine, Umm Al-Qura University, Makkah, SAU

**Keywords:** baricitinib, janus kinase inhibitors, nail lichen planus, systematic review, tofacitinib

## Abstract

Nail lichen planus (NLP) is a chronic immune-mediated inflammatory disorder and one of the most challenging forms of lichen planus to treat. Conventional therapies, including intralesional corticosteroids, systemic immunosuppressants, and topical agents, are often associated with high failure rates and significant adverse effects. Janus kinase inhibitors (JAKIs) have emerged as mechanistically rational therapeutic agents because of the central role of the interferon (IFN)-JAK-signal transducer and activator of transcription (JAK-STAT) pathway in lichenoid inflammation; however, their efficacy and safety in NLP have not yet been systematically evaluated. A systematic review was conducted by searching PubMed, Scopus, and Web of Science from database inception through March 2026 in accordance with the Preferred Reporting Items for Systematic Reviews and Meta-Analyses (PRISMA) 2020 guidelines. Eligible studies included patients with clinically and/or histopathologically confirmed NLP and studies reporting original patient-level data of any design. Risk of bias was assessed using the Joanna Briggs Institute (JBI) critical appraisal tools appropriate to each study design. A cumulative time-to-response model was constructed, and a random-effects meta-analysis of standardized mean change (SMC) in nail severity scores was performed where data permitted. Thirteen studies were included with a total of 25 patients. Interventions included oral and topical tofacitinib, baricitinib, abrocitinib, upadacitinib, and topical ruxolitinib. Clinically meaningful improvement was reported across all studies, with most responses occurring within three to seven months of treatment initiation. The pooled SMC was 1.65 (95% confidence interval (CI): 0-3.59), with substantial heterogeneity (I²=87.6%). No serious adverse events were reported. Overall, JAKIs appear to be effective and well tolerated in the short term for refractory NLP, supporting their potential role as rescue therapy in selected cases. However, the current evidence remains limited and heterogeneous, underscoring the need for prospective randomized controlled trials with standardized outcome measures and longer follow-up durations.

## Introduction and background

Nail lichen planus (NLP) is a rare chronic inflammatory nail disorder driven by immune-mediated mechanisms and is considered among the more difficult forms of lichen planus (LP) to manage therapeutically [1-3]. The condition can manifest with diverse nail abnormalities, including longitudinal ridging, nail plate thinning and brittleness, distal fissuring, onycholysis, lunular erythema, and in severe disease, dorsal pterygium formation. If left untreated or inadequately controlled, progressive nail damage may culminate in irreversible dystrophy or complete nail loss, substantially affecting both daily functioning and quality of life [4,5].

Diagnosis of NLP can be challenging because its clinical appearance often resembles other nail disorders, including nail psoriasis, trachyonychia, and onychomycosis. Consequently, delayed recognition and misdiagnosis are common. Nail biopsy with histopathological examination remains the gold standard for diagnosis and classically reveals a lichenoid inflammatory infiltrate involving the basal layer of the nail matrix [6-10].

Management of NLP continues to represent a significant therapeutic challenge, with no consistently effective treatment currently available. Topical agents such as corticosteroids and calcineurin inhibitors are frequently prescribed, although their effectiveness is often limited by inadequate penetration through the nail apparatus [2,4,7]. Intralesional corticosteroid therapy, especially with triamcinolone acetonide, is commonly regarded as a first-line option; however, treatment is often hindered by injection-associated pain, the requirement for multiple sessions, and variable therapeutic outcomes. Systemic therapies, including corticosteroids, methotrexate, acitretin, and cyclosporine, may achieve partial improvement, but their use is restricted by potential adverse events, inconsistent long-term responses, and recurrence after treatment withdrawal [10-13].

Recent advances in the understanding of LP immunopathogenesis have emphasized the importance of the Janus kinase-signal transducer and activator of transcription (JAK-STAT) pathway in disease development [3,6,9]. Interferon-gamma (IFN-γ)-mediated inflammation, along with downstream chemokines such as C-X-C motif chemokine ligand 10 (CXCL10), is believed to play a major role in sustaining chronic inflammation [2,3,6]. Additional cytokines, including interleukin-21 (IL-21), further enhance this immune response. Because JAK1 and JAK2 are integral mediators within this signaling cascade, they have emerged as promising therapeutic targets for LP and its nail manifestations [1-6].

These pathogenic insights have generated growing interest in Janus kinase inhibitors (JAKIs) as targeted treatment options. Both first-generation agents, including tofacitinib, baricitinib, and ruxolitinib, and newer selective JAK1 inhibitors such as abrocitinib and upadacitinib, have demonstrated encouraging results in various immune-mediated dermatologic conditions [1-13]. Nevertheless, current evidence regarding their use in NLP remains limited to case reports and small observational studies, and a comprehensive synthesis of available data is lacking. Therefore, this study was conducted to systematically evaluate the existing evidence regarding the efficacy and safety of JAKIs in patients with refractory NLP.

## Review

Methods

Study Design and Reporting Framework

This systematic review was conducted and reported in accordance with the Preferred Reporting Items for Systematic Reviews and Meta-Analyses (PRISMA) 2020 statement [14]. Adherence to these guidelines was intended to promote a transparent and systematic approach to study identification, screening, eligibility evaluation, and data synthesis.

Literature Search Strategy

A systematic and comprehensive literature search was performed across PubMed, Scopus, and Web of Science from their inception up to March 2026. The strategy combined controlled vocabulary (MeSH terms) with relevant free-text keywords pertaining to NLP and JAKIs, using Boolean operators to enhance both sensitivity and specificity of the search process (Table 1).

**Table 1:** Database search strategy used for identification of relevant studies This table outlines the Population, Intervention/Exposure, Comparison, and Outcome (PICO) framework used to define the eligibility criteria for studies included in this systematic review. The framework guided study selection and ensured alignment with the review objective of identifying behavioral and gynecologic risk factors associated with recurrent urinary tract infections in adult women.

Database	Search period	Search terms / Strategy	Filters applied
PubMed	Inception – March 2026	(“nail lichen planus” OR “ungual lichen planus” OR “nail LP”) AND (“Janus kinase inhibitor” OR tofacitinib OR baricitinib OR ruxolitinib OR abrocitinib OR upadacitinib OR JAK inhibitor)	No language restriction
Scopus	Inception – March 2026	TITLE-ABS-KEY (“nail lichen planus” AND “Janus kinase inhibitor”) OR TITLE-ABS-KEY (tofacitinib OR baricitinib OR ruxolitinib OR abrocitinib OR upadacitinib)	English only
Web of Science	Inception – March 2026	TS = (“nail lichen planus” AND “JAK inhibitor”) OR TS = (tofacitinib OR baricitinib OR ruxolitinib OR abrocitinib OR upadacitinib)	English only

In Scopus and Web of Science, searches were restricted to titles, abstracts, and keywords. No language restriction was applied in PubMed, while Scopus and Web of Science were limited to English-language publications due to database constraints.

Study Selection Process

All retrieved citations were imported into a reference management software, where duplicate records were identified and removed. Study selection was performed independently by two reviewers in a two-stage process, beginning with screening of titles and abstracts, followed by full-text assessment of potentially eligible articles.

Any discrepancies between the reviewers were resolved through discussion, and when consensus could not be reached, a third reviewer was consulted. To minimize the risk of missing relevant studies, the reference lists of all included articles and pertinent review papers were also manually searched for additional eligible publications.

Eligibility Criteria

Eligibility criteria were established using the Population, Intervention, Comparator, Outcomes (PICO) framework [15]. The study population comprised patients of any age or sex with clinically and/or histopathologically confirmed NLP, including both isolated disease and cases associated with cutaneous lichen planus. No restrictions were applied based on ethnicity, comorbid conditions, or disease severity. The intervention of interest included any JAKI, irrespective of its selectivity, formulation, dose, or route of administration. Agents such as tofacitinib, baricitinib, ruxolitinib, abrocitinib, upadacitinib, and other inhibitors targeting the JAK signaling pathway were considered (Table 2).

**Table 2:** PICO framework defining eligibility criteria for the systematic review This table outlines the Population, Intervention, Comparator, and Outcomes (PICO) framework used to define the eligibility criteria for study inclusion [15]. The population includes patients with nail lichen planus diagnosed clinically and/or histopathologically. The intervention of interest comprises all Janus kinase inhibitors (JAKIs), regardless of formulation, dosage, or route of administration. Comparators include conventional therapeutic approaches or no treatment; however, non-comparative studies were also eligible due to the limited available evidence. Outcomes of interest included measures of clinical response, validated severity scoring systems, quality-of-life indices, relapse rates, and adverse events.

Component	Description
Population (P)	Patients of any age or sex with clinically and/or histopathologically diagnosed nail lichen planus (isolated or associated with cutaneous lichen planus)
Intervention (I)	Any Janus kinase inhibitor (JAKI), including tofacitinib, baricitinib, ruxolitinib, abrocitinib, upadacitinib, and other agents, regardless of dose, route, or treatment duration
Comparator (C)	Conventional therapies (e.g., topical or intralesional corticosteroids, systemic corticosteroids, methotrexate, acitretin, cyclosporine) or no treatment; studies without comparators were also included
Outcomes (O)	Clinical improvement, validated severity scores (e.g., NALSI, tNLPSI), quality of life (DLQI), physician global assessment, time to response, relapse rate, and adverse events

Comparators included standard therapeutic approaches such as topical or systemic corticosteroids, retinoids, immunosuppressive agents, or no active treatment. Given the scarcity of comparative studies in this area and the predominance of observational evidence, studies without a comparator arm were also considered eligible for inclusion.

Outcomes of interest encompassed any reported measures of efficacy or safety, including clinical improvement, validated scoring systems such as the Nail Lichen Planus Severity Index (NALSI) and target Nail Lichen Planus Severity Index (tNLPSI), quality-of-life assessments (e.g., Dermatology Life Quality Index, DLQI), physician global assessment, time to clinical response, relapse rates, and adverse events.

Eligible study designs included case reports, case series, and observational cohort studies (retrospective or prospective) that provided original patient-level data.

Studies were excluded if they were review articles, editorials, conference abstracts lacking full-text availability, experimental animal studies, or in vitro research. Publications not specifically addressing nail lichen planus were also excluded, as were studies published in languages other than English.

Data Extraction

Data extraction was conducted independently by two reviewers using a standardized and pretested data collection form. The variables retrieved included study-level characteristics (author, publication year, country, and study design), patient demographics, disease duration, baseline clinical severity, number of nails involved, diagnostic approach, and prior or concomitant treatments. In addition, detailed information regarding JAKI therapy was recorded, including the specific agent used, dosage, route of administration, and treatment duration, along with all reported clinical outcomes.

In studies reporting outcomes at multiple follow-up points, all available time-point data were extracted to facilitate assessment of longitudinal treatment response patterns. Any inconsistencies between reviewers during data extraction were resolved through discussion, and when necessary, adjudication by a third reviewer was sought.

Risk of Bias Assessment

Risk of bias assessment was performed using the Joanna Briggs Institute (JBI) Critical Appraisal Checklists, selected according to the study design (case reports, case series, and cohort studies) [16].

Each included study was evaluated across relevant methodological domains, including the clarity of inclusion criteria, adequacy of diagnostic confirmation, completeness of demographic and clinical data reporting, use of consecutive case inclusion where applicable, transparency of outcome reporting, and appropriateness of statistical analysis when relevant.

The appraisal was conducted independently by two reviewers, and any disagreements were resolved through discussion until consensus was reached. Each item within the checklist was rated as “yes,” “no,” “unclear,” or “not applicable,” based on its relevance to the specific study design.

Data Synthesis and Statistical Analysis

A narrative synthesis was performed for all included studies, with results structured according to study design, patient characteristics, type of JAKI used, duration of therapy, and reported clinical outcomes. When adequate quantitative data were available, a meta-analysis was undertaken using a random-effects model to account for inter-study variability. Given the heterogeneity in outcome measurement scales across studies, the standardized mean change (SMC) was used as the primary effect size.

Statistical heterogeneity was evaluated using the I² statistic and Cochran’s Q test. Additionally, a cumulative time-to-response analysis was conducted by extracting reported onset times of clinical improvement across studies, enabling visualization of response patterns over the course of treatment. All statistical analyses were carried out using R software version 4.3 (R Foundation for Statistical Computing, Vienna, Austria).

Results

Study Selection

**Figure 1:**
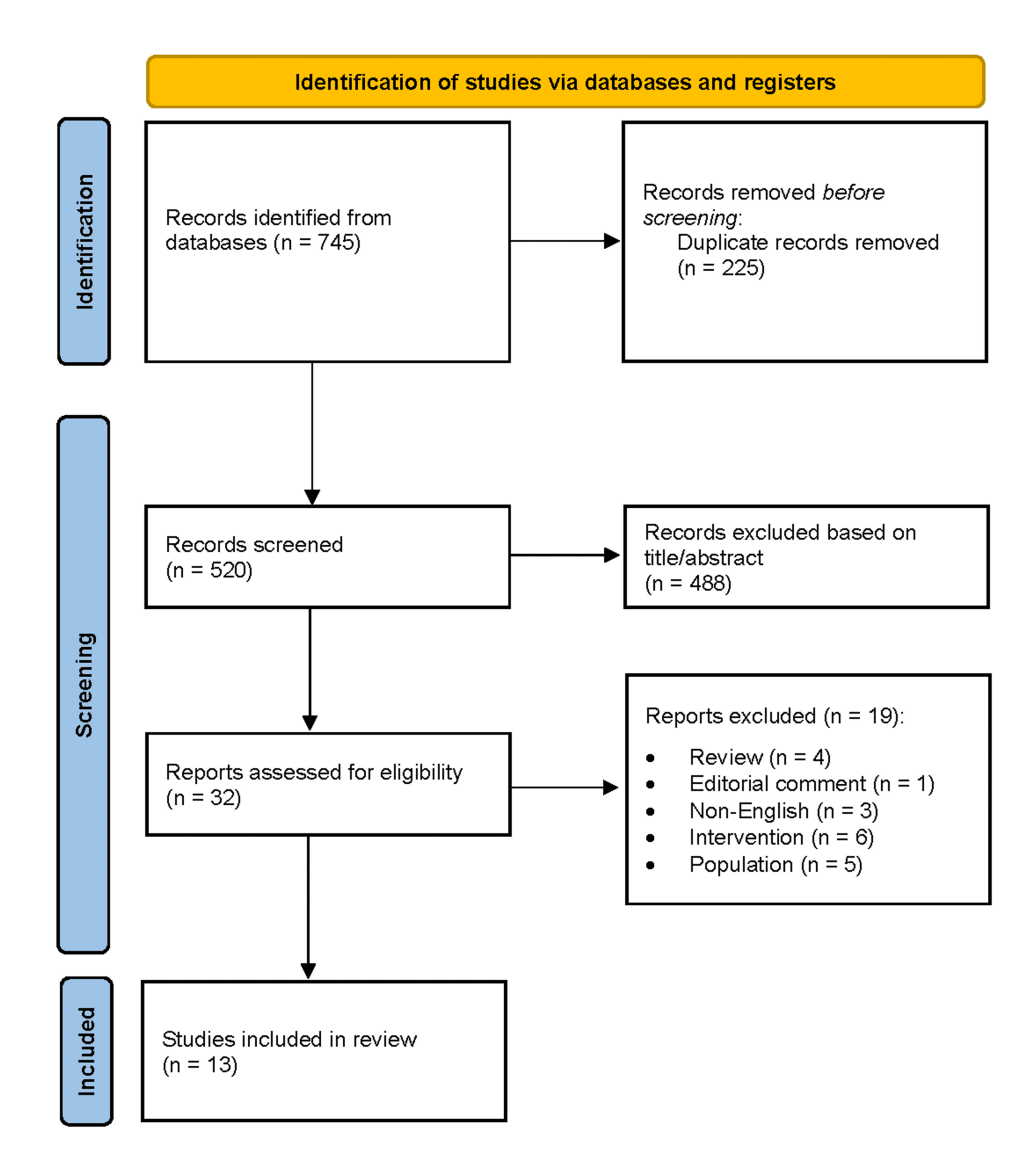
PRISMA flow diagram depicting the study selection process for the systematic review PRISMA (Preferred Reporting Items for Systematic Reviews and Meta-Analyses) flow diagram detailing the study selection process for this systematic review [14]. Following the identification of records through database searching and other sources, duplicates were removed, and the remaining records were screened. Full-text articles were assessed for eligibility, with exclusions documented along with reasons. Studies meeting the inclusion criteria were included in the final synthesis.

A total of 745 records were identified through database searching. After removal of 225 duplicates, 520 records remained, of which 488 were excluded based on title and abstract screening. Thirty-two full-text articles were assessed for eligibility, and 19 were excluded due to study type (reviews, n=4; editorial comment, n=1), non-English language (n=3), wrong intervention (n=6), or wrong population (n=5). Thirteen studies met the inclusion criteria and were included in the systematic review (Figure 1).

Study and Patient Characteristics

The 13 included studies, involved a total of 25 patients with biopsy-confirmed NLP treated with JAKIs [1-13]. The studies originated from China (n=7), Switzerland (n=3), India (n=1), Australia (n=1), and Iran (n=1), and were published between 2021 and 2026. Patient ages ranged from 10 to 60 years, with most cases reporting adult patients and a predominance of female patients in case reports, while cohort data showed a mean age of 25.3 ± 18.1 years with 77.7% male patients. Baseline disease severity was generally moderate-to-severe, with universal nail matrix involvement and occasional nail bed involvement. All patients had refractory disease with prior treatment failure, most commonly after topical corticosteroids, intralesional or systemic triamcinolone acetonide, methotrexate, acitretin, systemic corticosteroids, or topical tacrolimus (Table 3).

**Table 3:** Integrated baseline characteristics, treatment details, and outcomes of included studies evaluating JAKIs in NLP This table summarizes the combined baseline clinical characteristics, treatment regimens, and reported outcomes of patients with nail lichen planus (NLP) treated with Janus kinase inhibitors (JAKIs) across the included studies. Variables include study location, patient demographics (age and sex), disease duration prior to JAKI initiation, number of affected nails, baseline severity, nail matrix and nail bed involvement, comorbidities, prior treatments, and history of treatment failure. Treatment-related details include the specific JAK inhibitor used, dosage and route where available, and duration of therapy. Outcome measures include methods of assessing clinical response (e.g., visual assessment, validated severity scores such as NALSI and tNLPSI, physician global assessment, DLQI, and dermoscopy), along with diagnostic confirmation methods (primarily histopathology from nail biopsy where available). NR indicates data not reported in the original study. BID indicates twice daily administration. JAKI: Janus kinase inhibitor; NLP: nail lichen planus; NR: not reported; NALSI: Nail Lichen Planus Severity Index; tNLPSI: typical Nail Lichen Planus Severity Index; DLQI: Dermatology Life Quality Index; RA: rheumatoid arthritis.

Study	Country	Sample size	Age (years)	Sex	Disease duration (years)	Affected nails	Severity	Nail matrix	Nail bed	Comorbidities	Prior treatment	Drug	Duration (months)	Outcome assessment	Diagnosis method	Key conclusion
Luo et al. [1]	China	1	31	Female	3	20	Severe	Yes	Yes	None	Topical tacrolimus	Abrocitinib 100 mg daily	7	Clinical + dermoscopy	Biopsy histopathology	Highly effective and safe
Iorizzo et al. [2]	Switzerland	1	57	Female	NR	10	Severe	Yes	NR	Hypothyroidism, RA, alopecia universalis	Intralesional steroids, alitretinoin	Tofacitinib 5 mg BID	6	Visual clearance	Biopsy histopathology	Effective, no adverse events
Iorizzo et al. [3]	Switzerland	3	65	Male	NR	4	Moderate–severe	Yes	NR	None	Intramatricial triamcinolone injections	Topical tofacitinib 2% BID	3	NALSI, photographs	Biopsy-proven diagnosis	Significant improvement; pterygium and melanonychia persisted
			51	Male	NR	2	Moderate–severe	Yes	NR	None	Intramatricial triamcinolone injections	Topical tofacitinib 2% BID	3	NALSI, photographs	Biopsy-proven diagnosis	Significant improvement after 3 months
			59	Male	NR	4	Moderate–severe	Yes	NR	None	Intramatricial triamcinolone injections	Topical tofacitinib 2% BID	3	NALSI, photographs	Biopsy-proven diagnosis	Complete clearance in treated nails after 3 months
Thakur et al. [4]	India	9	25.3 ± 18.1 (range 11–60)	7 Male, 2 Female	NR	87 fingernails analyzed	Moderate–severe	Yes	Yes	NR	Multiple failed therapies	Oral tofacitinib	≥3	tNLPSI and 5-point PGA	Characteristic clinical features and/or histopathology	Significant improvement in tNLPSI and PGA scores from baseline to 3 months; effective and well-tolerated with no adverse effects reported
He et al. [5]	China	3	NR	NR	NR	NR	Severe classical NLP	Yes	NR	NR	Prior failed therapies and/or prior misdiagnosis common	Oral baricitinib	6	Physicians’ overall clinical impression	Longitudinal nail biopsy with histopathology	Great improvement after 6 months; no severe adverse events reported
			30s	Male	2	Multiple	Severe classical NLP	Yes	Yes	None	Topical glucocorticoid encapsulation therapy and oral acitretin	Oral baricitinib 4 mg daily	6	Physicians’ overall clinical impression	Longitudinal nail biopsy with histopathology	Near-complete remission after 6 months
			39	Female	>1	Multiple	Moderate classical NLP	Yes	Yes	None	Topical antifungal therapy	Oral abrocitinib 100 mg daily	6	Physicians’ overall clinical impression	Longitudinal nail biopsy with histopathology	Near-complete remission with minimal relapse during follow-up
He et al. [6]	China	1	30s	Male	2	Multiple	Severe	Yes	Yes	None	Acitretin, topical therapy	Baricitinib 4 mg daily	6	Visual + DLQI	Biopsy	Successful control
He et al. [7]	China	1	39	Female	>1	Multiple	Moderate–severe	Yes	Yes	None	Antifungal therapy	Abrocitinib 100 mg daily	12	Visual + DLQI + tNLPSI	Biopsy	Safe and effective
Huang et al. [8]	China	1	41	Female	2	10	Severe	Yes	No	None	Steroids, tacrolimus	Tofacitinib 5 mg BID	6	Visual clearance	Biopsy	Effective refractory option
Shakoei et al. [9]	Iran	1	51	Female	5	20	Severe	Yes	NR	None	Multiple systemic therapies	Tofacitinib 5 mg BID	6	Clinical photos	Biopsy	Effective and safe
Sheng et al. [10]	China	1	10	Male	3	Multiple	Severe	Yes	Yes	Vitiligo	NR	Ruxolitinib cream	10	Clinical + dermoscopy	Clinical/dermoscopy	Safe pediatric option
Chim et al. [11]	Australia	1	43	Male	3	10	Severe	Yes	NR	None	Steroids	Baricitinib 3.4 mg BID	13	Visual clearance	Clinical	Effective; relapse after cessation
Zhao et al. [12]	China	1	33	Female	12	20	Severe	Yes	Yes	None	Multiple systemic therapies	Upadacitinib 15 mg daily	6	NALSI score	Biopsy	Effective; mild recurrence
Pünchera et al. [13]	Switzerland	1	60s	Female	1	NR	NR	NR	NR	None	Multiple systemic therapies	Baricitinib 4 mg daily	NR	NR	NR	Highly effective, well-tolerated

Risk of Bias Assessment

Risk of bias was assessed using the JBI Critical Appraisal Checklists. Most domains (D1-D3, D6-D9) were adequately reported across studies. Consecutive inclusion (D4) and complete inclusion (D5) were unclear in multi-patient studies (Table 4).

**Table 4:** Risk of bias assessment of included studies using Joanna Briggs Institute critical appraisal tools This table presents the methodological quality and risk of bias assessment of all included studies evaluating Janus kinase inhibitors (JAKIs) in nail lichen planus (NLP), using the Joanna Briggs Institute (JBI) critical appraisal checklists [16]. Each study was evaluated across key methodological domains, including clarity of inclusion criteria, validity of diagnostic methods, consecutive and complete inclusion of participants, adequacy of demographic and clinical reporting, completeness of outcome reporting, and appropriateness of statistical analysis where applicable. Responses for each domain are categorized as “Yes,” “No,” “Unclear,” or “Not applicable” depending on study design. Case reports and case series were not assessed for statistical analysis or consecutive sampling when not relevant to the study design. Overall risk of bias was classified qualitatively as low or moderate based on completeness and clarity of reporting across domains.

Study	Clear inclusion criteria	Valid diagnostic methods	Consecutive inclusion	Complete inclusion	Clear patient demographics	Clear clinical history	Clear reporting of outcomes	Appropriate statistical analysis	Overall risk of bias
Luo et al. [1]	Yes	Yes	Not applicable	Yes	Yes	Yes	Yes	Not applicable	Low
Iorizzo et al. [2]	Yes	Yes	Not applicable	Yes	Yes	Yes	Yes	Not applicable	Low
Iorizzo et al. [3]	Yes	Yes	Unclear	Unclear	Yes	Yes	Yes	Not applicable	Moderate
Thakur et al. [4]	Yes	Yes	Unclear	Unclear	Yes	Yes	Yes	Yes	Moderate
He et al. [5]	Yes	Yes	Unclear	Unclear	Yes	Yes	Yes	Yes	Moderate
He et al. [6]	Yes	Yes	Not applicable	Yes	Yes	Yes	Yes	Not applicable	Low
He et al. [7]	Yes	Yes	Not applicable	Yes	Yes	Yes	Yes	Not applicable	Low
Huang et al. [8]	Yes	Yes	Not applicable	Yes	Yes	Yes	Yes	Not applicable	Low
Shakoei et al. [9]	Yes	Yes	Not applicable	Yes	Yes	Yes	Yes	Not applicable	Low
Sheng et al. [10]	Yes	Yes	Not applicable	Yes	Yes	Yes	Yes	Not applicable	Low
Chim et al. [11]	Yes	Yes	Not applicable	Yes	Yes	Yes	Yes	Not applicable	Low
Zhao et al. [12]	Yes	Yes	Not applicable	Yes	Yes	Yes	Yes	Not applicable	Low
Pünchera et al. [13]	Yes	Unclear	Not applicable	Yes	Yes	Yes	Unclear	Not applicable	Moderate

Discussion

This systematic review synthesizes the available evidence on JAKIs in refractory NLP, a condition that remains difficult to manage due to its chronic inflammatory behavior, frequent diagnostic overlap with other nail disorders, and limited response to conventional therapies. Across 13 included studies involving 25 patients, JAK inhibition was consistently associated with clinical improvement, suggesting a potential therapeutic role in selected refractory cases [1-13].

Pathophysiological Rationale for JAK Inhibition

The observed clinical responses are supported by the immunopathogenesis of NLP, which is primarily driven by cytotoxic T-cell-mediated injury of the nail matrix. IFN-γ plays a central role in amplifying this inflammatory response through activation of the JAK-STAT signaling cascade, leading to downstream chemokine expression (including CXCL10) and sustained lymphocytic infiltration.

JAK1 and JAK2 are key mediators of this pathway, and their inhibition disrupts signal transduction at an upstream level, thereby attenuating inflammatory propagation. This mechanistic framework provides biological plausibility for the consistent therapeutic responses observed across both non-selective and selective JAKIs in the included studies [1-13]. The similar clinical outcomes across different agents also suggest that blockade of this central signaling axis, rather than drug-specific effects, is likely responsible for disease control.

Clinical Efficacy Across Studies

Despite variability in study design, treatment regimens, and outcome reporting, all included studies demonstrated some degree of clinical improvement following JAKI therapy [1-13]. Tofacitinib was the most frequently reported agent and consistently showed meaningful improvement in nail morphology, often within three to seven months of initiation [2,4,8,9]. In several cases, near-complete resolution of nail dystrophy was reported, even in patients with long-standing, treatment-refractory disease.

Baricitinib also demonstrated favorable outcomes in multiple reports, with rapid improvement in nail changes and functional outcomes [6,11,13]. However, relapse after discontinuation in at least one case suggests that sustained suppression of disease activity may require ongoing therapy in some patients [11].

Selective JAK1 inhibitors, including abrocitinib and upadacitinib, were associated with significant improvement in individual cases, with stabilization of disease activity and reduction in severity scores [1,7,12]. One report also noted recurrence following dose reduction, indicating a potential dose-dependent effect on disease control [12].

Topical JAKIs, including tofacitinib cream and ruxolitinib cream, demonstrated efficacy in localized and pediatric disease, offering a potential alternative approach that minimizes systemic exposure while maintaining clinical benefit [3,10].

Treatment Response Dynamics

A consistent observation across studies was a delayed but progressive clinical response. Initial improvement was generally observed within the first one to three months, while maximal response typically occurred between three and seven months of therapy [1-13]. This timeline aligns with the slow growth rate of the nail unit and the time required for regeneration following suppression of matrix inflammation.

Cumulative response rates ranging from approximately 0.75 to 0.90 further support the potential effectiveness of JAK inhibition in refractory NLP, although these estimates should be interpreted cautiously given the small sample sizes and heterogeneous outcome measures [1-13].

Safety and Tolerability

Across all included studies, JAKIs were generally well tolerated, with no serious adverse events reported during follow-up [1-13]. This favorable short-term safety profile is notable given the systemic immunomodulatory effects of these agents.

However, the limited duration of follow-up (three to 13 months) restricts meaningful conclusions regarding long-term safety. Potential concerns such as infection risk, metabolic changes, hematologic abnormalities, and rare adverse events cannot be adequately assessed in the current evidence base.

Limitations

This review is limited by the predominance of low-level evidence, as most included studies were case reports and small case series, which are inherently prone to publication bias favoring positive outcomes. The absence of randomized controlled trials, lack of blinding, and absence of comparator arms in most studies further limit the strength of the conclusions. Follow-up durations were heterogeneous and generally short (three to 13 months), limiting assessment of long-term efficacy, relapse rates, and safety. In addition, variability in diagnostic approaches raises the possibility of misclassification bias, particularly given the known risk of prior misdiagnosis in NLP. Outcome measures and treatment regimens were also inconsistent across studies, including differences in JAKI type, dosing, and route of administration, which contributed to substantial heterogeneity and limited comparability across reports.

## Conclusions

In conclusion, this systematic review suggests that JAKIs may offer a promising therapeutic option for NLP, with generally favorable short-term clinical responses and an acceptable safety profile in refractory cases. The observed effects are consistent with the proposed immunopathological rationale for targeting the JAK-STAT pathway in inflammatory nail disease. However, the current evidence remains limited and heterogeneous, and definitive treatment recommendations cannot yet be made. Well-designed randomized controlled trials with standardized outcome measures, adequate follow-up durations, and comparative evaluation of different agents are required to better define efficacy, safety, and long-term outcomes.
